# Three-dimensional reconstruction of single-cell chromosome structure using recurrence plots

**DOI:** 10.1038/srep34982

**Published:** 2016-10-11

**Authors:** Yoshito Hirata, Arisa Oda, Kunihiro Ohta, Kazuyuki Aihara

**Affiliations:** 1 Institute of Industrial Science, The University of Tokyo, 4-6-1 Komaba, Meguro-ku, Tokyo 153-8505, Japan; 2 Department of Life Sciences, Graduate School of Arts and Sciences, The University of Tokyo, 3-8-1 Komaba, Meguro-ku, Tokyo 153-8902, Japan

## Abstract

Single-cell analysis of the three-dimensional (3D) chromosome structure can reveal cell-to-cell variability in genome activities. Here, we propose to apply recurrence plots, a mathematical method of nonlinear time series analysis, to reconstruct the 3D chromosome structure of a single cell based on information of chromosomal contacts from genome-wide chromosome conformation capture (Hi-C) data. This recurrence plot-based reconstruction (RPR) method enables rapid reconstruction of a unique structure in single cells, even from incomplete Hi-C information.

Through the development of measurement techniques, we are getting the glimpse that chromosomal structures in individual cells may be differentially organized to exhibit distinct genome and chromatin activities. Such cell-to-cell variability might be an important aspect of cell physiology during differentiation or cellular responses. Genome-wide chromosome conformation capture (Hi-C) analysis^1^,^2^,^3^ has been developed to estimate the three-dimensional (3D) chromosome structure, allowing greater accuracy of detection for local chromosomal contacts in a single cell^4^,^5^. However, single-cell Hi-C is normally based on limited information of DNA contacts owing to the limited recovery of nuclear DNA from a single cell, while in regular Hi-C data, one can also employ read counts which enable more detailed estimation. Reconstruction of the 3D chromosome structure from such incomplete data is often difficult and requires long calculation times, even with high-performance computers.

In a mathematical sense, reconstruction of the 3D chromosome structure from Hi-C data can be considered a problem of geometry. Therefore, many *ad hoc* computational methods have been proposed^6^,^7^ for reconstructing the 3D chromosome structure from Hi-C data. However, such methods have several disadvantages. First, there is no mathematical support that guarantees the correctness of the estimation. Second, such methods are often formulated as stochastic optimizations, and thus, their solutions are not unique. Finally, we sometimes need to use additional information other than the contact information for every pair of DNA segments.

Here, to overcome such difficulties, we propose a new mathematical method using recurrence plots^8^,^9^ for reconstruction of the 3D chromosome structure. This method, referred to as recurrence plot-based reconstruction (RPR) method, enables the reconstruction of a unique 3D chromosome structure solely from contact map information obtained by Hi-C analysis. In addition, the RPR method is applicable, even to low-coverage chromosomal contact Hi-C data from a single cell. The algorithm can indeed reconstitute high-resolution whole chromosomal structures, even in the case of a single mammalian cell with a large genome.

## Methods

**Figure 1:**
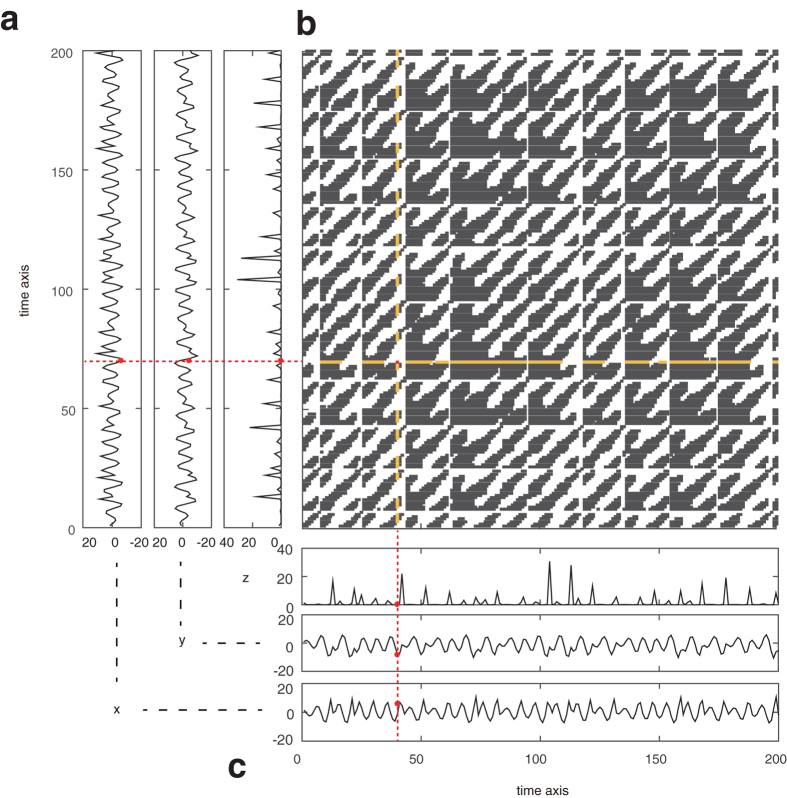
Example of a recurrence plot. Panels a and c show the same three dimensional time series. Panel b shows its recurrence plot. Namely, both axes show the same time axis. If a three-dimensional distance between a pair of sets of coordinates for the corresponding times is small, then we plot a point at the corresponding place in the recurrence plot.

A recurrence plot^8^,^9^ is a tool of nonlinear time series analysis for visualizing temporal patterns within a time series. It is a two-dimensional plot, and both axes show the same time axis. In a recurrence plot, one can show whether or not the states corresponding to each pair of times are close to each other in state space (see Fig. 1). Mathematically, a recurrence plot R can be defined as follows:


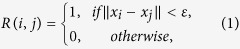


where *x*_*i*_ is a state corresponding to time *i*, and ε is a predefined threshold. We plot a point at (*i, j*) when *R*(*i, j*) = 1 and nothing there if *R*(*i, j*) = 0 (see Fig. 1). Thus, the recurrence plot allows for display of the closeness between every pair of points, similar to Hi-C data. Therefore, if we regard chromosomal positions as time points in a time axis, we can naturally treat Hi-C data as a recurrence plot.

Several techniques^10^,^11^,^12^ have been developed for the recovery of a rough shape of the original time series solely from the information of its recurrence plot. However, we have chosen to focus on the RPR method we previously proposed in 2008^11^ because (i) it works, even if the original time series is multivariate, and (ii) we have proven a theorem^13^ that the metric space recovered using our prior method^11^ is equivalent to the original Euclidean metric under mild conditions. In addition, the RPR method is known to be rather robust, even if we change the definition of the closeness^14^. Therefore, we used the method to reconstruct the 3D chromosome structure from Hi-C data.

**Figure 2:**
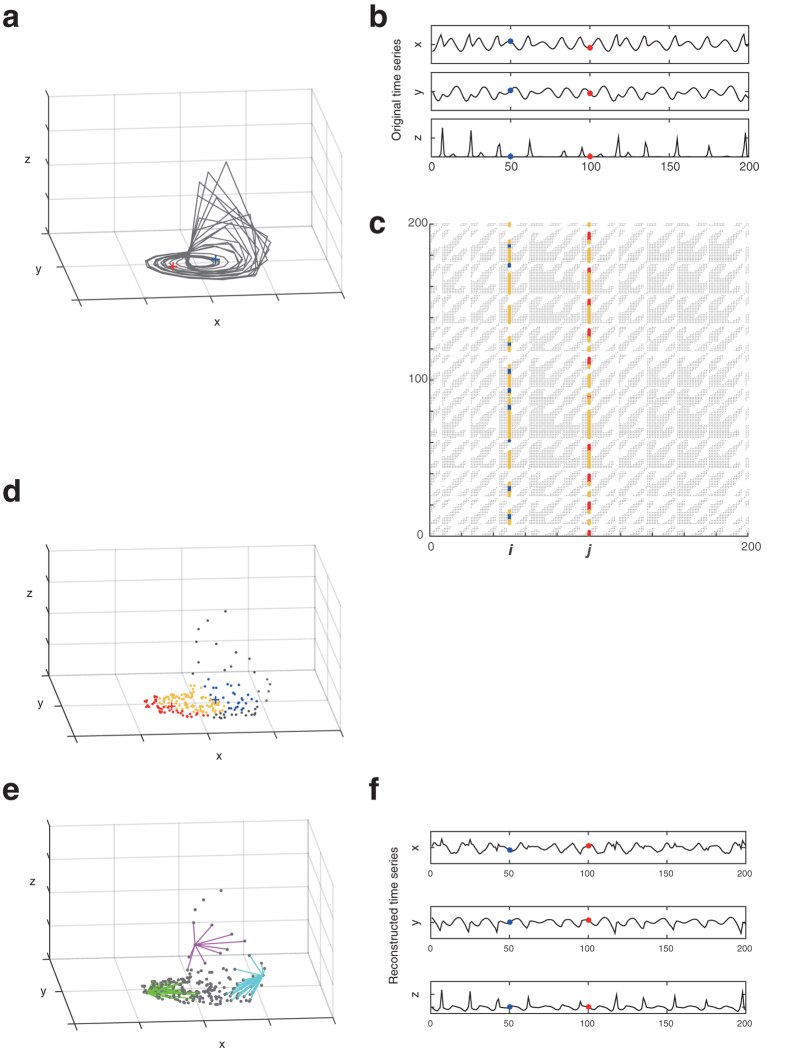
How to reconstruct the original time series from a recurrence plot. Panel a shows the original three-dimensional attractor of the Rössler model. Panel b shows the corresponding time series. Panel c shows its recurrence plot. In Panel b, we especially highlight the *i*th and the *j*th time points with blue and red, respectively. In Panel c, we show the common vertical time indices when the states are close to those at both the two time points *i* and *j* with yellow, while vertical time indices are plotted in blue or red when the states are close only to that at time *i* or that at time *j*, respectively. In Panel d, the corresponding time indices are located in the three-dimensional space with the same color as Panel c. In addition, the *i*th and *j*th points are shown with the blue plus and the red plus, respectively. Between these *i*th and *j*th points, we define a distance defined by Eq. (2). For more generally, local neighbors are connected as shown in magenta, green, and cyan links in Panel e. Although we keep the visibility by showing three neighbor examples of time points in Panel e, eventually all points are connected with their neighbors during the reconstruction process for the original time series and we generate a weighted graph; because this model is in the three dimensional space, the weighted graph could be embedded possibly in the three dimensional space as shown in Panel e. Then, we obtain the shortest distances between every pair of time points and thus vertices on this weighted network. Lastly we apply the multidimensional scaling. If we plot the most three major components, we can reproduce the three-dimensional time series as shown in Panel f, which look roughly similar to the original time series shown in Panel b.

**Figure 3:**
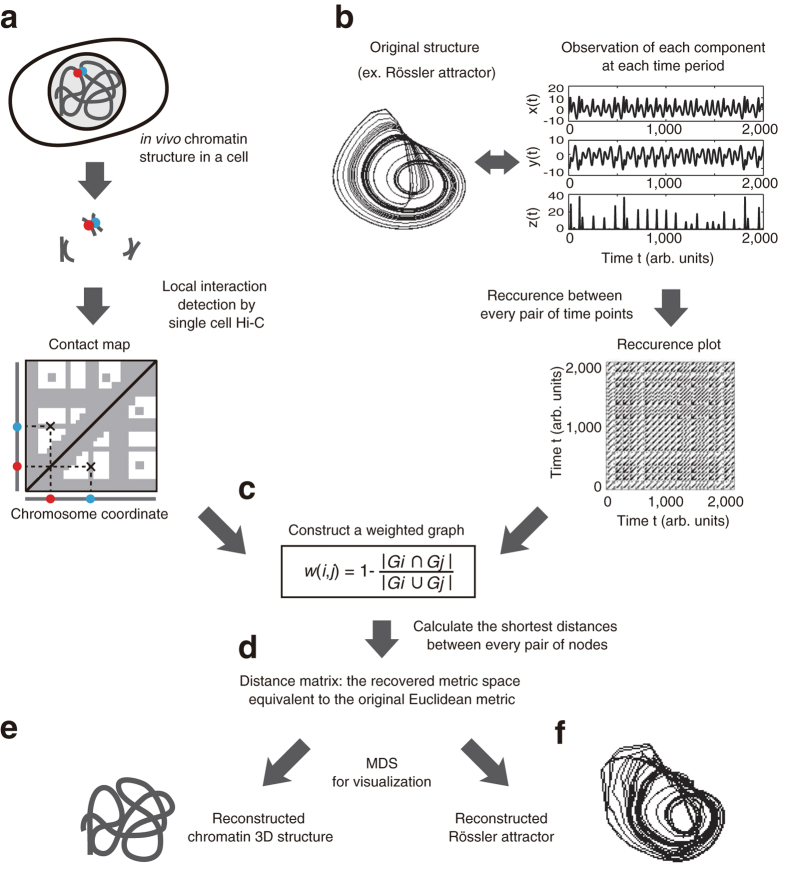
Overview of the RPR reconstruction for three-dimensional chromosomal structure. Single-cell Hi-C data represent the spatial closeness of two parts of chromosomes to reveal the *in vivo* chromatin structure (**a**), and a recurrence plot shows the spatial closeness for states of every pair of time points to visualize time series data (**b**). According to a previously published method^11^, we first constructed a weighted graph within which neighbors were connected by paths with the length shown in (**c**), and we then obtained the shortest distance between every pair of nodes (**d**). Application of multidimensional scaling (MDS) enables us to reconstruct the 3D structures of chromosomes (**e**) and the Rössler attractor (**f**).

The proposed procedure according to our previous method^11^ can be roughly summarized as shown in Figs 2 and 3. First, we converted Hi-C data into a weighted graph (see Figs 2 and 3). In this graph, each node corresponds to a part of a chromosome, and we connect two nodes if the Hi-C data show that the corresponding two parts are located in close proximity (beneath the defined threshold). In addition, we assign the following local distance to the edge between the two nodes *i* and *j*:


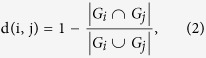


where *G*_*i*_ = {*k*|*R*(*k, i*) = 1} is a set of indices plotted in the *i*th column of the recurrence plot and thus the corresponding Hi-C data (see Supplementary Texts A to understand intuitively that Eq. (2) gives us the original local distance between points *i* and *j* when they are close to each other). Second, we obtain the shortest distance by the Dijkstra method^15^ for every pair of nodes to obtain a distance matrix of global distances. Third, we used multidimensional scaling^16^ for visualizing the information of the distance matrix. By choosing the three largest eigenvalue components of the multidimensional scaling, we can reconstruct the 3D chromosome structure (see Figs 2 and 3). This RPR method is developed especially for low coverage data such as single-cell Hi-C analysis. The key point of this algorithm is that it treats the contact maps as binary without coverage information. Namely, if two corresponding segments of chromosomes are detected as neighbors at least once within a Hi-C dataset, we assign 1 to the corresponding two elements of the contact map, while if the two segments are not neighbors, then we assign 0 to the corresponding elements of the contact maps. Thus, this definition of the binary contact map is universal for both single Hi-C data and regular Hi-C data and does not need a threshold for the binarization. The threshold *ε* for the recurrence plot is defined intrinsically by the experimental limits of detectable contacts by the sparse Hi-C method as well as the regular Hi-C method. When the coverage depth is too high, the regular Hi-C data may be pre-processed to reduce the number of counts. But, presumably this pre-processing will not be necessary because the proposed RPR method can be used even in the case where as high as 70% of elements for the binary contact map are 1 (see ref. 11).

A necessary condition for our previous method^11^ is that all nodes are connected with each other in the weighted graph, i.e., there is a path connecting every pair of nodes. To fulfill this necessary condition, we used a previously described method^12^ to identify successive parts of chromosome as neighbors and declare that *R*(*i, i* + 1) = *R*(*i* + 1, *i*) = 1 when the parts corresponding to *i* and (*i* + 1) are on the same chromosome. Using this technique, we can ensure that the proposed reconstruction is possible in most cases. If, instead, we modify *d*(*i, j*) as a constant value when regions *i* and *j* have a contact or |*i*−*j*| ≤ 1, the RPR method becomes more similar to the method of Paulsen *et al*.^17^.

Thus, as similar to reconstructing the three dimensional structure of strange attractors for deterministic chaos as shown in Fig. 2, we reconstruct the three-dimensional structure of chromosomes by regarding a contact map of Hi-C data as a recurrence plot. Figure 3 shows the similarity between the two reconstruction processes.

## Results

Single-cell Hi-C cannot report all the chromosomal interactions within a cell. In addition, sequencing data sometimes include errors. We therefore tested by toy models whether the RPR method is tolerant to the contamination of false positive noise and/or to the lack of information, as is often found in actual Hi-C data. Here we used the Lorenz model^18^ and Rössler model^19^ as examples of three-dimensional objects because they are in the three-dimensional space as similarly to chromosomes and thus the mathematical background for reconstructing the three-dimensional structures from their recurrence plots is similar to the three dimensional reconstruction of chromosomes from their contact map. Supplementary Figure 1 demonstrates the results using the Lorenz model^18^ and the Rössler model^19^, which are popular deterministic chaos models, with 1% bit flips and 90% random loss of information on the closeness. Correlations between the original data and the noisy data were kept as high as 0.70–0.92 in the test. Furthermore, datasets with only 0.2% of closest interaction information were used to mimic the single-cell Hi-C data. Then, we could visually recognize the topological similarities between the original shapes (Supplementary Figure 1a,f) and the reconstructions (Supplementary Figure 1e,j) using the limited information, suggesting that this method is sufficiently tolerant.

**Figure 4:**
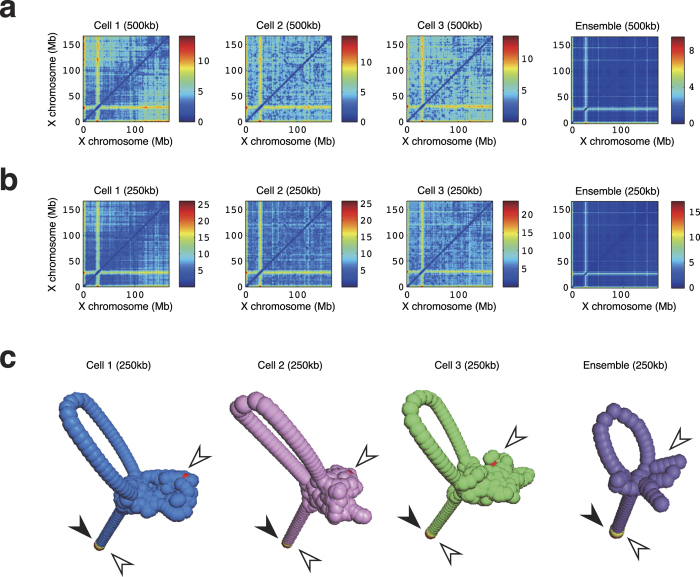
Example of 3D reconstruction of X chromosomes. (**a,b**) Heat maps of distance matrices of global distances calculated by the RPR method from Hi-C interaction data for three single cells (cells 1, 2, and 3) and the ensemble at a resolution of 500 (**a**) or 250 kb (**b**). (**c**) Reconstructed 3D chromosome X structure for cells 1, 2, 3 and the ensemble at 250-kb resolution. The red and yellow dots with black and open arrowheads indicate the positions of telomeres and centromeres, respectively.

Finally, we show an example of estimation for X chromosomes in male mouse T_H_1 cells from Hi-C data for different single cells and from an ensemble (regular) Hi-C data (Fig. 4)^5^. We first reconstructed distance information as distance matrices to reconstruct the 3D structure of each X chromosome at two different resolutions (500 and 250 kb; Fig. 4a,b). Between the two reconstructed distances at 500- and 250-kb resolutions, we obtained correlation coefficients of 0.9598, 0.9492, and 0.9481 for cells 1, 2, and 3, respectively. Therefore, our reconstructions seemed to be consistent and reasonable. In addition, at the 250-kb resolution, we obtained correlation coefficients of 0.7802, 0.7456, and 0.7405 between the reconstructed distributions of cell 1 and cell 2, those of cell 1 and cell 3, and those of cell 2 and cell 3, respectively. When we applied multidimensional scaling^16^ to the reconstructed distances, we obtained 3D chromosome structures (Fig. 4c) with a common feature, i.e., one of the X chromosome telomeres and an open loop were protruding from a cluster of other parts of the X chromosome. The topological structure which we found coincided well with that in Fig. 3A of Paulsen *et al*.^17^, where the same Hi-C data were used to reconstruct the three-dimensional structures.

**Figure 5:**
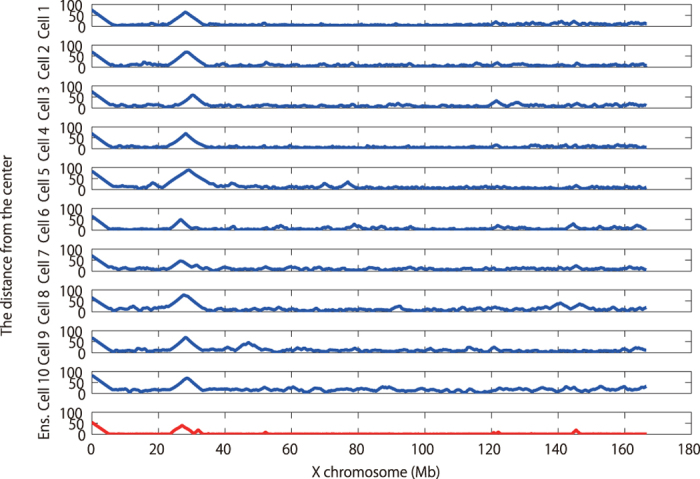
Cell-to-cell variability of predicted locations of chromatin opening and looping for chromosome X. We show how each segment of X chromosome is far from the cell center in our 50 kbp-resolution reconstructions for Cells 1–10 and the ensemble.

We further applied the RPR method to cells 4–10 of ref. 5 in 250 kbp resolution (Supplementary Figure 2) as well as cells 1–10 of ref. 5 in 50 kbp resolution (see Fig. 5 and Supplementary Figure 3). Despite some common features such as an open loop at a position of 30 Mb in all cells, careful inspection reveals some cell-to-cell variability in the open loops seen at positions of 70 or 145 Mb. We further confirmed that application of the RPR method to the ensemble Hi-C data could reproduce the similar features (Figs 4 and 5, and Supplementary Figure 3).

**Figure 6:**
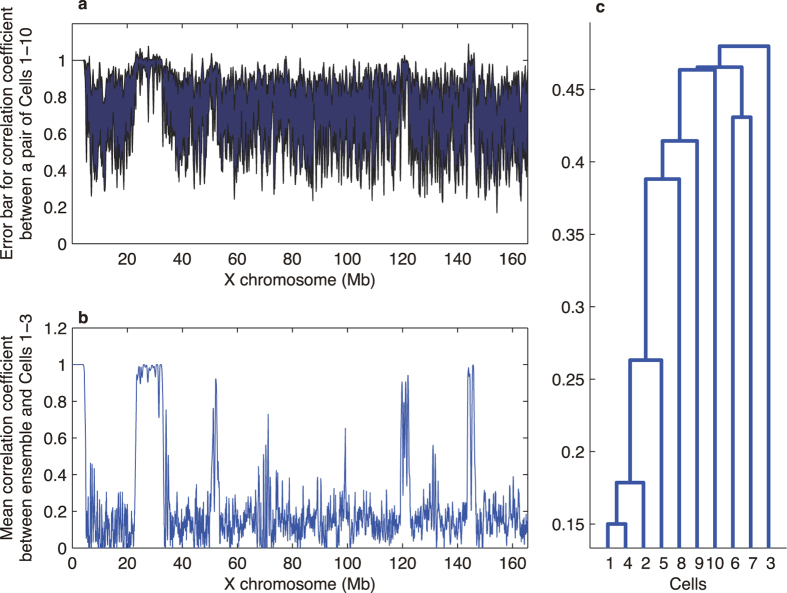
Cell-to-cell variability and similarity among the analyzed cells. Panel a shows the error bar corresponding the one sigma range for the correlation coefficient representing the similarity of local structure of our reconstruction for each pair of individual cells. Panel b shows the similarity between the reconstruction for the ensemble and those of individual cells. Namely, where the individual reconstructions are similar to that for the ensemble, the cell-to-cell variability is small, while if the individual reconstructions do not look similar to that for the ensemble, then cell-to-cell variability is high. Panel c shows the dendrogram showing which cell structure is more similar to which cell. Namely, Cell 1 and Cell 4 are most similar in their structures compared to the other cells.

There is some cell-to-cell variability among these cells. To demonstrate the cell-to-cell variability, we focused on the similarity and difference between the local structures in the scale of 1 Mb by taking “3D correlation coefficients” (see Supplementary Texts B for the details of the calculations). Figure 6a shows the correlation of local structures between every combinational pair of 10 individual cells, while Fig. 6b shows the correlation between the ensemble and each individual cell. The cell-to-cell variability is small when the reconstruction for each cell agreed with that of the ensemble (see Fig. 6a,b). This agreement means that from the analysis of the mass of cells, the 3D structure from ensemble Hi-C remarkably shows the common features that were observed within most of the ten individual cells of single cell Hi-C. On the other hand, structural fluctuation was observed at other parts of chromosomes where the topology for each cell is different from the ensemble. This structural fluctuation suggests the potential of single-cell Hi-C analysis to show the chromosome structure variability that cannot be seen in the ensemble Hi-C. Indeed, we can show how the topological structures are similar and different among individual cells in Fig. 6c.

The Th1 ensemble Hi-C data and single-cell Hi-C data were acquired from the GEO database (http://www.ncbi.nlm.nih.gov/geo/, accession GSE48262; GSM1173492 for the ensemble Hi-C data, and from GSM1173493 to GSM1173502 for the 10 single cell Hi-C data).

## Discussions

The RPR method is qualitatively different from other ways^2^,^7^,^20^,^21^,^22^,^23^,^24^,^25^,^26^,^27^,^28^ of chromosomal structural reconstruction (except for that of Paulsen *et al*.^17^) in terms of usage of single-cell Hi-C data without any consideration on read counts^29^. The method of Paulsen *et al*.^17^ is reported as an analysis appropriate for single-cell Hi-C data. While the method of Paulsen *et al*.^17^ only uses information of whether two segments of chromosomes are close or not for connecting an edge by just using a constant local distance, the RPR method can reflect information from distant segments when connecting two segments by an edge: it refines how close they are by reconstructing the local distance by Eq. (2), which is strongly related to the Jaccard coefficient^30^ (see also Supplementary Texts A). Thus, the RPR method potentially enables us to reproduce the metric space and thus rationally estimate three-dimensional chromosome structures especially from very low coverage Hi-C data such as single-cell Hi-C. For overcoming the rough approximation at each local distance, the method of Paulsen *et al*.^17^ intentionally puts less weights on the distantly located pairs to reduce the influence of noise since a long path composed by many edges is less reliable. On the other hand, the RPR method does not have currently special mechanisms to reduce such noise since the RPR method is tolerant to noise and lack of information as shown in Supplementary Figure 1: it is because some small fractions of bit flips or missing bits due to such noise cause small differences in the evaluations of Eq. (2) and thus the topological structures reconstructed. Hence, if we combine the noise reducing mechanism of Paulsen *et al*.^17^ for long paths with the weighted graph of the RPR method, one may be able to reproduce the 3D structure of chromosomes more accurately. This direction is an open remaining problem.

Another important advantage of the RPM method is that it provides a unique three-dimensional reconstruction. This is because (i) the proposed method coverts a contact map to a distance matrix in a deterministic way, and (ii) the classical multidimensional scaling implemented in Matlab gives a unique configuration for a spatial arrangement of points given their distance matrix.

In addition, the RPR method has other advantages in comparison with the conventional methods^2^,^7^,^17^,^20^,^21^,^22^,^23^,^24^,^25^,^26^,^27^,^28^ such as methods by Lense *et al*.^7^ and Paulsen *et al*.^17^. First, the RPR method only needs the information of the contact map, whereas the method by Lense *et al*.^7^ requires additional information such as the frequencies of pairs or read counts, hence the RPR method is suitable for Hi-C data with a few read counts. Second, the proposed method is based on mathematical proofs^13^,^14^ and seems to work well with larger datasets. For example, the RPR method functioned well, even for a large-scale dataset with over 10,000 points^3^, whereas the example with Lense’s method handled only up to 1,000 points^7^. Third, the proposed method could be carried out within a reasonable time on an easily available computer. For example, it takes less than an hour to reconstruct the 3D chromosome structure for a whole set of chromosomes of a single cell at 250-kb resolution if we replace the Dijkstra method with the Johnson method^31^, even when we use a conventional computer with 2 × 2.66 GHz 6-Core Intel Xeon and 64 GB memory with codes implemented on Matlab (Supplementary Codes). To make this comparison reasonable and fair, we used the codes provided by the link of ref. 17, and reproduced the structure of X chromosome in 50 kbp resolution using the same above computer. Then we found that the method of ref. 17 needed 764 seconds, while the proposed RPR method needed 157 seconds and reproduced the typical topology appropriately (Here we replaced the Dijkstra method with the Johnson^31^ method implemented in Matlab’s graphallshortestpaths function. This function for finding the shortest path lengths for all pairs of nodes is originally used in the codes of ref. 17 and thus we decided to employ it for speeding up; See Supplementary Table 1 and Supplementary Figure 4 for further comparisons). Thus, the proposed method is expected to provide a breakthrough for the reconstruction of the 3D chromosomal structure from Hi-C data.

## Additional Information

**How to cite this article**: Hirata, Y. *et al*. Three-dimensional reconstruction of single-cell chromosome structure using recurrence plots. *Sci. Rep.* **6**, 34982; doi: 10.1038/srep34982 (2016).

## Supplementary Material

Supplementary Information
