# Supplementary material for: Three-dimensional reconstruction of single-cell chromosome structure using recurrence plots
Source: Sci Rep. 2016 Oct 11;6:34982. doi: 10.1038/srep34982 (PMC5057099; doi:10.1038/srep34982)
Supplement: Supplementary Information [file srep34982-s1.pdf]

Supplementary Information

Three-dimensional reconstruction of single-cell chromosome structure using recurrence plots

Yoshito Hirata, Arisa Oda, Kunihiro Ohta, & Kazuyuki Aihara

Supplementary Texts

Supplementary Figures 1-4

Supplementary Table 1

Supplementary Codes

Supplementary Texts

A. Intuitive explanation on why Eq. (2) can reproduce the local distances approximately

Here is our intuitive explanation on why the local distance reconstructed by Eq. (2) is proportional to the local distance between two neighbors in the original three-dimensional space. Suppose that (i) points are distributed uniformly, (ii) the local distance between two neighbors in the original space is r and (iii) the threshold used for obtaining a recurrence plot is ε (see Supplementary Figure 5). Then, Eq. (2) for the two points in the three dimensional space can be derived as

$$1-\frac{(\text{Volume for region B})}{(\text{Total volume for regions A, B, and C})}=1-\frac{\frac{4}{3}\pi\varepsilon^3-2\int_0^{\frac{1}{2}r}\pi(\varepsilon^2-x^2)dx}{\frac{4}{3}\pi\varepsilon^3+2\int_0^{\frac{1}{2}r}\pi(\varepsilon^2-x^2)dx}. \quad \text{(S1)}$$

By calculating Eq. (S1), we have

$$\frac{r\left\{1-\frac{r^2}{3\varepsilon^2}\right\}}{\frac{1}{3}\varepsilon\left\{1+\frac{r}{\varepsilon}-\frac{r^3}{3\varepsilon^3}\right\}}. \quad \text{(S2)}$$

If we assume $r \ll \varepsilon$, it is easy to see that Eq. (S2) is proportional to r. By assembling this type of local distances, we reproduce global distances and thus the topology of chromosomes. See Refs. 13 and 14 for the detail.

B. How to compare local structures in our reconstruction: "3D correlation coefficients"

In Figure 6, we compared local structures of our reconstructions by first obtaining distance matrices and second obtaining their correlation coefficients. By following this procedure, we can compare local structures which are invariant under rotation, reflection, shift, and change of size.

Suppose that we have a series $\{x_i \in R^3 | i = 1,2,\dots,N\}$ of points in the three-dimensional space. For this series, an N-by-N distance matrix D can be obtained as $D(i,j) = \|x_i - x_j\|$, where $\| \ \|$ shows the Euclidean norm, namely the square root of the sum of squares for the components, $D(i,j) = \sqrt{(x_i - x_j)^T (x_i - x_j)}$. Here, we consider a transformed series of $\{x_i\}$ by

$$y_i = cAx_i + b, \qquad \text{(S3)}$$

where $A \in R^{3\times3}$ is a square matrix with $|A| = 1\ or -1$ representing the rotation and/or the reflection, $b \in R^3$ corresponds to the shift in the space, and $c > 0$ is a coefficient for the change of the size. We define that the distance matrix for $\{y_i | i = 1,2,\dots,N\}$ as Δ. Thus, each component of Δ can be written as

$$\Delta(i,j) = \sqrt{\left(y_i - y_j\right)^T\left(y_i - y_j\right)} = \sqrt{\left(cAx_i + b - cAx_j - b\right)^T\left(cAx_i + b - cAx_j - b\right)}$$

$$= \sqrt{\left(cAx_i - cAx_j\right)^T\left(cAx_i - cAx_j\right)} = c\sqrt{\left(Ax_i - Ax_j\right)^T\left(Ax_i - Ax_j\right)}$$

$$= c\sqrt{\left(x_i - x_j\right)^T A^T A\left(x_i - x_j\right)} = c\sqrt{\left(x_i - x_j\right)^T\left(x_i - x_j\right)} = cD(i,j), \qquad \text{(S4)}$$

which implies $\Delta = cD$ as a whole. Thus, if Eq. (S3) holds and we take the correlation coefficient between D and Δ, it becomes 1. Namely, by obtaining distance matrices followed by taking their correlation coefficient, we can identify whether their structures are similar or not when we remove the freedoms of the rotation, the reflection, the shift, and the change of size.

We compared local structures for a pair of reconstructed chromosomes in a similar way to Ref. 32. Namely, we prepared the window of 1Mb in the axis of X chromosome. Then, we slid it by 0.05 Mb repeatedly from the left arm to reach the right arm. At each position of the window, we applied the above two-step comparison to produce Figures 6a and 6b. Namely, if the local structures of the two X chromosomes at the corresponding window position are similar in 1Mb wide, then the value for the correlation coefficient becomes close to 1, while if their local structures at the corresponding window position in 1 Mb wide are more different, then the value for the correlation coefficient becomes smaller.

In addition, we used the dendrogram for representing which cell is closer to which cell in the topological structure. We used the correlation coefficient between the distance matrices for the whole X chromosome reconstructions of every pair of 10 cells to produce the dendrogram.

Additional Reference

32. Fukino, M., Hirata, Y. & Aihara, K. Coarse-graining time series data: Recurrence plot of recurence plots and its application to music. *Chaos* **26**, 023116 (2016).

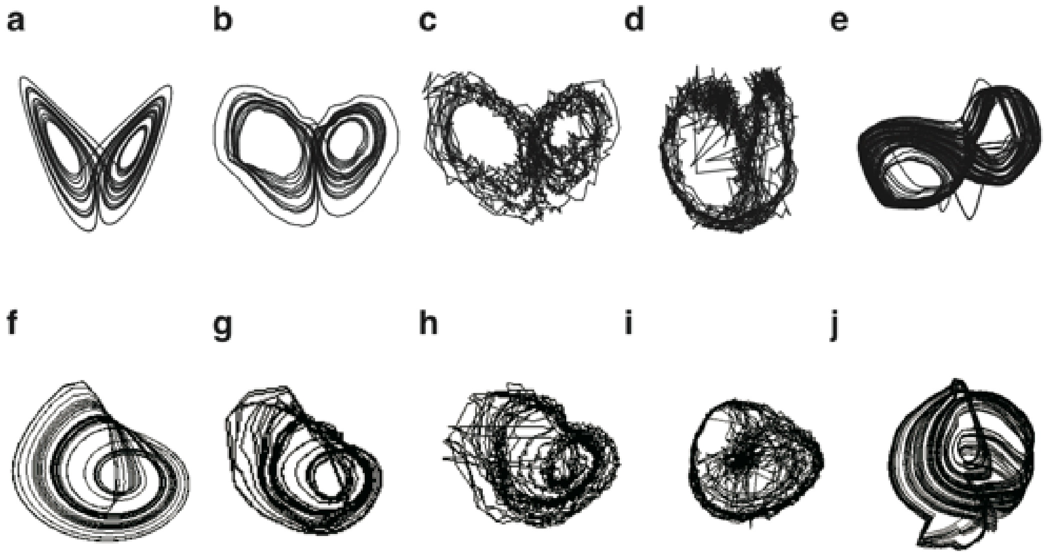

Supplementary Figure 1 | Attractors for two models of deterministic chaos and their reconstructions from recurrence plots

(a) and (f) are for the original shapes of the Lorenz and Rössler models. (b)–(e) and (g)–(j) are the reconstructions by recurrence plots using our previously published method[11]. (b) and (g) are from the original recurrence plots, where only 20% of points retain information. (c) and (h) show reconstructions of models that lose information of 90% of the plotted points. In (d) and (i), 1% of the points are bit flipped. (e) and (j) are the same as (b) and (g), except that only 0.2% of points retain information but with a longer time series to mimic the case of Hi-C data of a single cell.

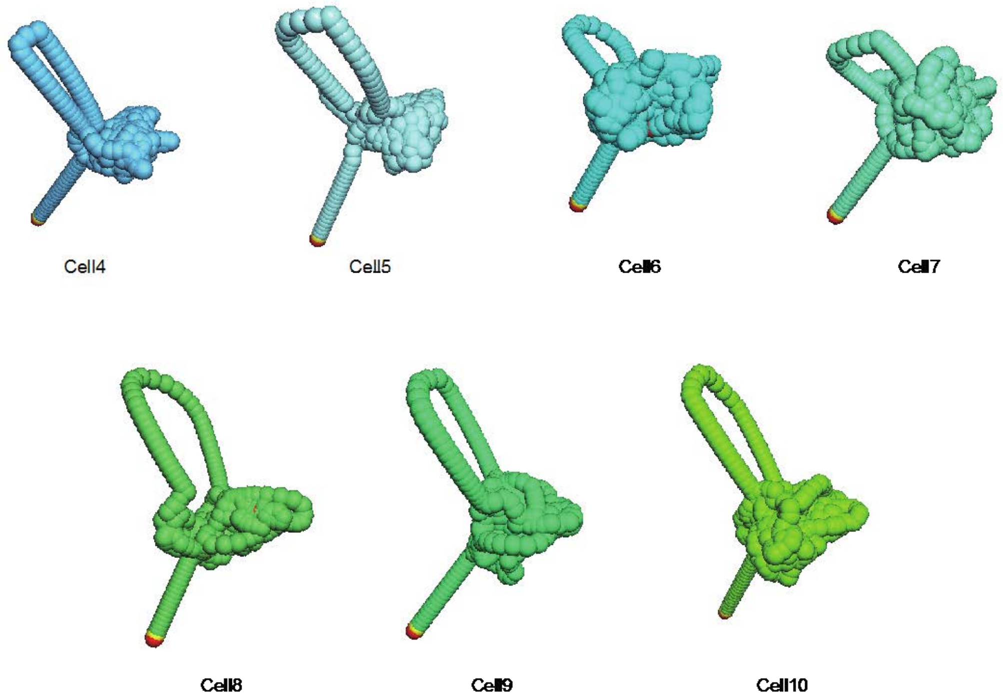

Supplementary Figure 2 | Three dimensional chromosome reconstructions for single cells in 250kbp resolution

Three-dimensional reconstructions for Cells 4-10 based on single-cell Hi-C data.

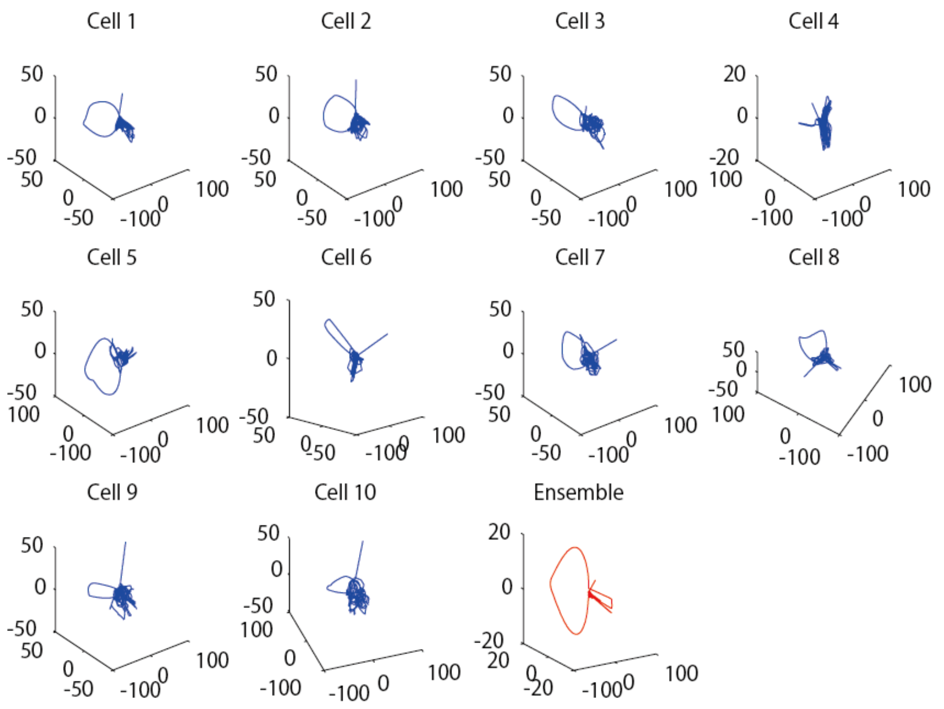

Supplementary Figure 3 | Three-dimensional reconstruction of X chromosome in 50kbp resolution

Each panel corresponds to Cells 1-10 and the ensemble Hi-C data.

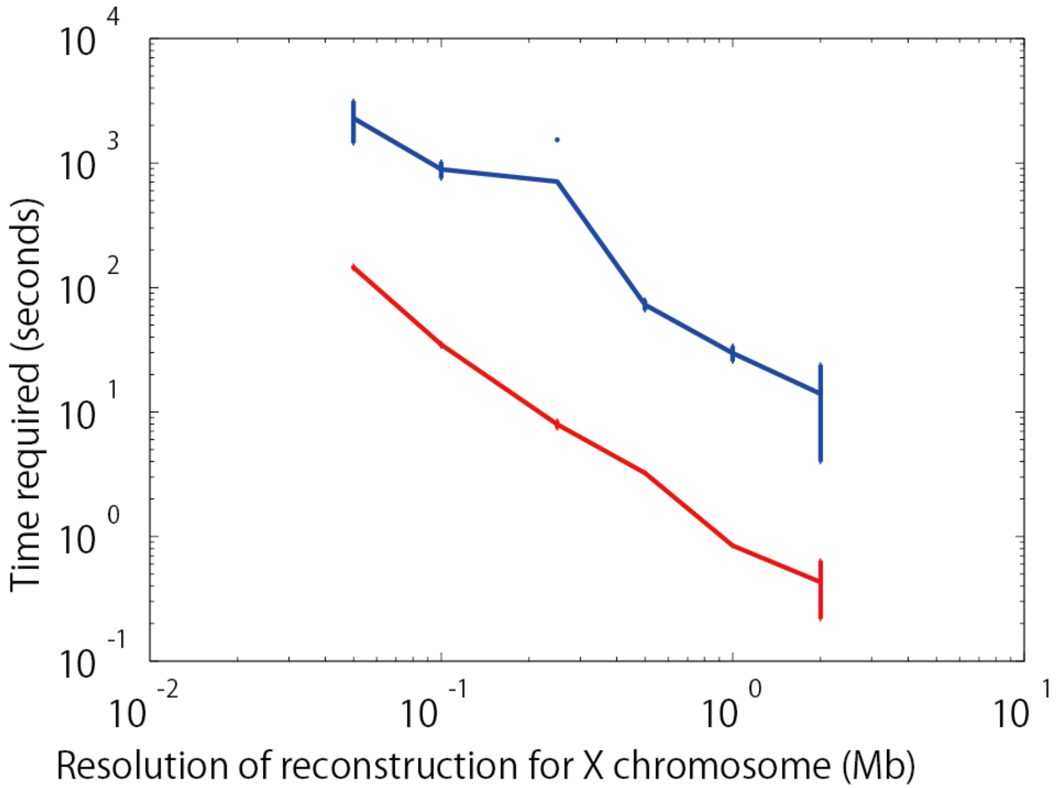

Supplementary Figure 4 | Comparison in computational time required for the method of Paulsen et al. (blue) and the proposed RPR method (red).

The error bar was obtained for reconstructing three-dimensional structures for Cells 1 and 2. The computational time was obtained using a computer with 8 core CPU (Intel(r) Xeon(r) E5-4640 at 2.40GHz) and 1TB memory.

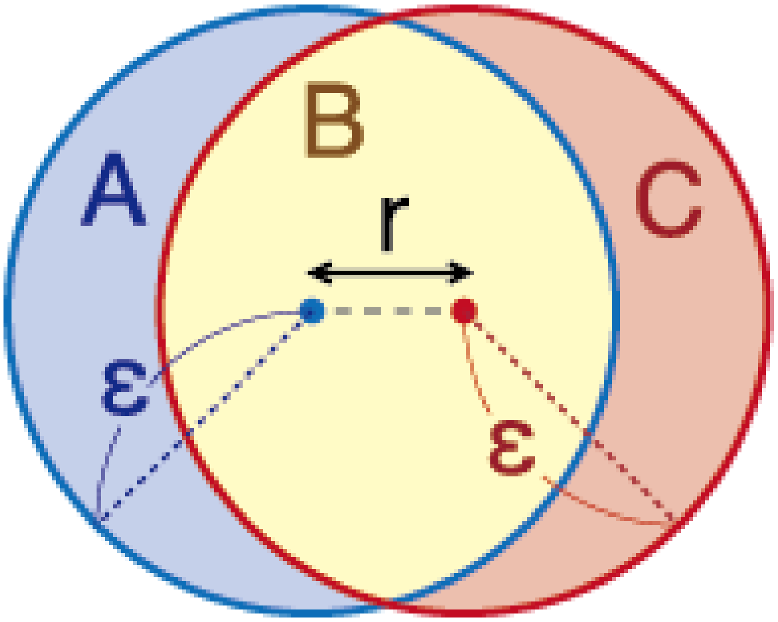

Supplementary Figure 5 | Schematic graph for two neighboring points for an intuitive explanation. There are two three-dimensional balls of radius ε defining the neighbors for the two points separated by r. Region B is the intersection between the two balls. Region A is the region where points belong to the left ball but not to the right ball, while in Region C, points belong to the right ball but not the left ball. If points are distributed uniformly, then Eq. (2) becomes proportional to the original local distance r between the two points. See the Supplementary Texts A for the detailed calculation.

Supplementary Table 1 | Comparisons of reconstructions using the method of Appendix B between the method of Paulsen et al. and the proposed RPR method when the reconstruction resolution was varied. Reconstructions for Cells 1 and 2 were compared for the method of Paulsen et al. (MBO) and the RPR method. We excluded the entries for which we obtained NaN for MBO for the comparisons. We found the tendency that the two reconstructions for MBO and RPR become more similar when the resolution becomes more coarse (the 4th and 5th columns), which means that when the resolution is fine, one of the reconstructions is not appropriate. When we compare the two reconstructions using the same method, the two reconstructions by MBO show lower correlations than those by RPR (see the 3rd and 2nd columns). Thus, these correlations imply that the reconstructions by MBO become worse if we try to use the finer resolution.

Resolution	RPR1-RPR2	MBO1-MBO2	RPR1-MBO1	RPR2-MBO2
50kbp	0.8289	0.1537	0.3219	0.2314
100kbp	0.7777	0.1867	0.4051	0.3295
250kbp	0.6855	0.1979	0.5580	0.3066
500kbp	0.5970	0.1935	0.6627	0.3898
1Mbp	0.5373	0.1823	0.7843	0.5058
2Mbp	0.5737	0.2248	0.7795	0.5880

Supplementary Codes

```
function [y3,dists] = reproduce3dstructure(rp1,ns)

%
%   rp1: contact map (binary sparse matrix showing 1 when a pair of segments has a contact or 0 otherwise)
%   ns: The number of segments for each chromosomes
%         The sum sum(ns) should be equal to size(rp1,1) and size(rp1,2).
%   y3: three dimensional reconstruction
%

n = size(rp1,1);

for ii = 1:size(ns,2)
     for jj = (sum(ns(1:(ii-1)))+1):(sum(ns(1:(ii-1)))+ns(ii))
             rp1(jj,jj) = 1; % Each segment is close to itself.
      end

      for jj = (sum(ns(1:(ii-1)))+1):(sum(ns(1:(ii-1)))+ns(ii)-1)
             rp1(jj,jj+1) = 1; % Consecutive segments are also close to each other.
             rp1(jj+1,jj) = 1;
      end
end

dists = calculatedistanceongraph2c3b(rp1); % Apply the proposed method using recurrence plot

[y,e] = cmdscale(dists); % Apply the multidimensional scaling

y3 = y(:,1:3); % Extract the three most major components


function [dists,D] = calculatedistanceongraph(T)

n = size(T);
n1 = n(1);
n2 = n(2);
```

```
D = sparse(n1,n1);

for i = 1:n1
    i
    temp = find(T(i,:) == 1); % Find a set of time indices
    D(i,i) = 0;
    for j = (i+1):n1
        if sum(temp == j) > 0
            temp2 = find(T(j,:) == 1); % Find another set of time indices
            temp3 = intersect(temp,temp2); % Find their intersection.
            temp4 = unique([temp temp2]); % Find their union.
            temp5 = 1-(size(temp3,2)/size(temp4,2)); % Calculate the local distance defined by Eq. (2).
            D(i,j) = temp5; % Assign the local distance so that
            D(j,i) = temp5; % the distance matrix becomes symmetric.
        end
    end
end

dists = graphallshortestpaths(D,'directed',false); % Find all of the shortest distances on the graph
```
